# Protein microcrystallography using synchrotron radiation

**DOI:** 10.1107/S2052252517008193

**Published:** 2017-08-08

**Authors:** Masaki Yamamoto, Kunio Hirata, Keitaro Yamashita, Kazuya Hasegawa, Go Ueno, Hideo Ago, Takashi Kumasaka

**Affiliations:** a Advanced Photon Technology Division, RIKEN SPring-8 Center, 1-1-1 Kouto, Sayo-cho, Sayo-gun, Hyogo 679-5148, Japan; b Precursory Research for Embryonic Science and Technology (PRESTO), Japan Science and Technology Agency, 4-1-8 Honcho, Kawaguchi, Saitama 332-0012, Japan; c Protein Crystal Analysis Division, Japan Synchrotron Radiation Research Institute, 1-1-1 Kouto, Sayo-cho, Sayo-gun, Hyogo 679-5198, Japan

**Keywords:** protein microcrystallography, multi-point data collection, multi-crystal data collection, serial synchrotron crystallography

## Abstract

The progress in X-ray microbeam applications using synchrotron radiation is beneficial to structure determination from macromolecular microcrystals such as small *in meso* crystals. However, the high intensity of microbeams causes severe radiation damage, which worsens both the statistical quality of diffraction data and their resolution, and in the worst cases results in the failure of structure determination. Even in the event of successful structure determination, site-specific damage can lead to the misinterpretation of structural features. In order to overcome this issue, technological developments in sample handling and delivery, data-collection strategy and data processing have been made. For a few crystals with dimensions of the order of 10 µm, an elegant two-step scanning strategy works well. For smaller samples, the development of a novel method to analyze multiple isomorphous microcrystals was motivated by the success of serial femtosecond crystallography with X-ray free-electron lasers. This method overcame the radiation-dose limit in diffraction data collection by using a sufficient number of crystals. Here, important technologies and the future prospects for microcrystallography are discussed.

## Abstract 2

Recent developments in protein microcrystallography using synchrotron radiation are reviewed, including the use of a high-flux microbeam, consideration of radiation damage, sample-handling techniques, and data-collection strategies and their automation.

## 1. The introduction of microcrystallography   

Three-dimensional structures of biological macromolecules are becoming increasingly important in modern biology and medicine, especially with the rapid growth in demand for the investigation of functionally important targets such as membrane proteins. Atomic structures of macromolecules are indispensable for determining biological functions. Macromolecular crystallography (MX) is an important high-resolution structure-analysis method for biological macromolecules. The development of synchrotron beamlines and modern molecular-biology techniques have continuously improved MX. Recently, with the advent of microfocus beamlines and X-ray free-electron lasers (XFELs), which can utilize a microbeam of 10 µm or less, microcrystallography targeting crystals of about 10^9^ unit cells, around 10 × 10 × 10 µm or less in size, has been developed. Here, we focus on the history of microcrystallography, which was enabled by technological progress such as the emergence of microbeams.

In the 1990s, the increase in the number of macromolecular structures deposited in the Protein Data Bank (Berman *et al.*, 2003 ▸) was accelerated by the introduction of synchrotron radiation (SR) and the development of highly sophisticated structure-analysis software (Evans *et al.*, 2011 ▸). In September 2016, the Protein Data Bank contained over 120 000 structures. SR has the remarkable feature of providing highly brilliant X-ray beams at a wide range of wavelengths (Helliwell & Mitchell, 2015 ▸). The brilliant beams achieved using SR have improved data quality and decreased the crystal size required for structure determination. The wavelength variability of SR makes it easy to obtain experimental phases using anomalous diffraction (Hendrickson, 1999 ▸, 2014 ▸). SR MX beamlines now provide the majority of X-ray structures that are deposited every year (Evans *et al.*, 2011 ▸).

During the progress of MX using SR, the targets of structure analysis have evolved to be more challenging and scientifically important (Helliwell & Mitchell, 2015 ▸; Gruner & Lattman, 2015 ▸). The major difficulty in structure determination of these challenging targets is obtaining crystals with a suitable size and sufficient diffracting power for crystallo­graphic data collection. In the case of membrane proteins, structure determination has been dramatically accelerated by the development of a new crystallization method using lipidic mesophases (Caffrey, 2003 ▸) in the mid-1990s. Because *in meso* crystals tend to be very small (∼10 µm), the demand for high-quality data collection from microcrystals has increased. The high flux density and small focus size of microbeams make them suitable for data collection (Caffrey *et al.*, 2012 ▸; Caffrey, 2015 ▸). Kobilka and coworkers used a microbeam to collect data from *in meso* microcrystals of G protein-coupled receptors (GPCRs) as challenging targets and determined a number of important structures such as that of the β_2_-adrenergic receptor (Rasmussen *et al.*, 2007 ▸; Rosenbaum *et al.*, 2007 ▸; Cherezov *et al.*, 2007 ▸). The researchers that achieved this accomplishment were subsequently awarded the Nobel Prize in Chemistry in 2012 (Lefkowitz, 2012 ▸; Kobilka, 2012 ▸).

To cope with targets such as GPCRs, microfocus beamlines dedicated to protein microcrystallography with a focal beam size smaller than a few tens of micrometres and a flux density of over 10^10^ photons µm^−2^ s^−1^ at the sample position have recently been constructed at many third-generation SR facilities (Smith *et al.*, 2012 ▸). Nonetheless, the use of a high-flux microbeam can easily cause serious radiation damage even for cryocooled microcrystals (Moukhametzianov *et al.*, 2008 ▸; Sanishvili *et al.*, 2011 ▸). Holton & Frankel (2010 ▸) theoretically estimated that a complete dataset with a signal-to-noise ratio of 2 at 2 Å resolution would be obtainable from a cryocooled perfectly spherical lysozyme crystal with a diameter of 1.2 µm before the dose reached the radiation-damage limit. However, there is still a discrepancy between this theoretical limit and experimental results because the noise arising from background scattering was not included and the assumption of a perfect detector was used in the calculation. Therefore, to fully utilize microfocus MX beamlines, protein microcrystallo­graphy needs to be generalized by developing and upgrading the light source and experimental apparatus, such as the diffractometer and the area detector. Moreover, novel data-collection strategies must be introduced (Owen *et al.*, 2016 ▸). Further development of protocols for more efficient and precise data collection from multiple microcrystals is needed.

Multicrystal data collection (Kendrew *et al.*, 1960 ▸; Clemons *et al.*, 2001 ▸; Liu *et al.*, 2011 ▸) has recently been revived in microcrystallography (Brockhauser *et al.*, 2012 ▸; Zander *et al.*, 2015 ▸); even in these cases, the number of crystals used for structure determination usually remained relatively small at about several dozen crystals. However, continuous development of the technology now allows the use of a larger number of smaller crystals for data collection; for example, fast readout detectors increase the number of datasets that can be collected in an appropriate amount of beamtime, a sample changer allows rapid sample exchange without manual operation, and the development of sophisticated software makes it easy to merge multiple datasets.

In addition to the progress in microcrystallography using SR, the ultrabrilliant femtosecond X-ray pulses of X-ray free-electron lasers (XFELs) as a new X-ray source have led to a novel approach for data collection called serial femtosecond crystallography (SFX; Chapman *et al.*, 2011 ▸; Schlichting, 2015 ▸). Data collection in SFX entails the collection of many thousands of images from many thousands of crystals. These crystals are serially delivered to the X-ray beam by the flow of a microcrystal suspension (injector-based SFX) or the translation of microcrystal arrays fixed on a thin-film substrate (fixed-target SFX). The advantage of XFELs for microcrystallography is that they allow data collection without radiation damage; Neutze *et al.* (2000 ▸) theoretically predicted that a single XFEL pulse of a few tens of femtoseconds would allow the collection of a still diffraction image before destruction of the protein by Coulomb explosion, which was later proved by Chapman *et al.* (2011 ▸). The performance of SFX has been demonstrated by the successful structure determination of challenging targets (Kang *et al.*, 2015 ▸; Batyuk *et al.*, 2016 ▸; Zhou *et al.*, 2015 ▸; Colletier *et al.*, 2016 ▸).

The success of SFX motivated researchers to establish serial crystallography using synchrotron microfocus MX beamlines (serial synchrotron crystallography; SSX) by combining a high-intensity microbeam and a rapid detector. One approach is fixed-target SSX, in which large number of images are collected by two-dimensional raster scanning from multiple crystals loaded on nylon-loop or thin-film substrates (Gati *et al.*, 2014 ▸; Coquelle *et al.*, 2015 ▸). Another approach utilizes a continuous flow of a microcrystal suspension using a capillary (Stellato *et al.*, 2014 ▸) or injectors in combination with a high-viscosity medium (Botha *et al.*, 2015 ▸; Nogly *et al.*, 2015 ▸).

**Figure 1:**
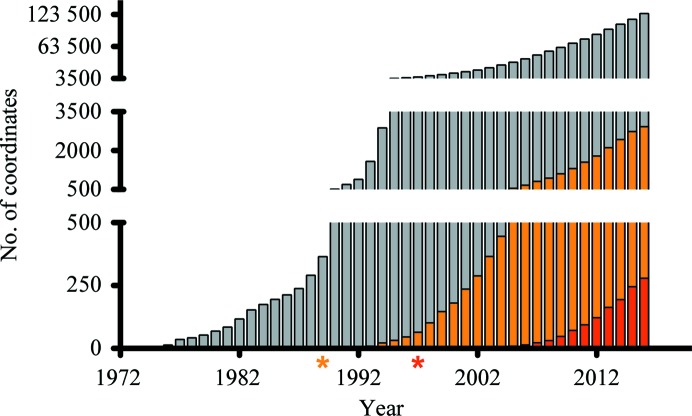
The accumulation of deposited coordinates in the Protein Data Bank. The total numbers of deposited coordinates per year are shown as grey bars. The counterparts for all membrane proteins and human membrane proteins are shown as orange and red bars, respectively. The asterisks show the year of the first structure deposition of a membrane protein (orange) and a human membrane protein (red). In the decade after the first structure, the number of deposited coordinates of membrane proteins grew in an exponential manner. This exponential growth implies the contribution of technical breakthroughs such as the usage of recombinant DNA for protein production in the case of soluble proteins in the early 1980s.

The current growth rate in the accumulated structures of membrane proteins is almost equal to that of soluble protein structures in the 1990s (Fig. 1 ▸). Important targets for MX are human membrane proteins owing to their direct relevance to human diseases and drug discovery, but available structures of these still remain limited. In an attempt to determine the structures of such samples, various developments in the technology and methodology for microcrystallography are being pursued (Moraes, 2016 ▸). In this review, recent developments in microcrystallography using SR are summarized.

## 2. Requirements for protein microcrystallography   

### 2.1. A highly brilliant microbeam   

**Figure 2:**
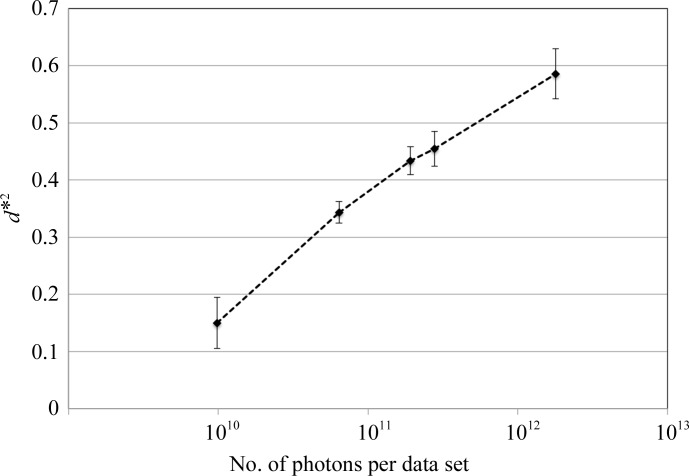
Relationship between the number of incident X-ray photons and the resolution achieved. These datasets were collected on beamline BL32XU at SPring-­8 from nine thaumatin crystals with sizes of ∼100 µm. From each crystal, five datasets, each consisting of 100° of rotation, were collected from the same crystal volume with different numbers of incident photons, 9.8 × 10^9^ (0.1 MGy), 6.4 × 10^10^ (0.2 MGy), 1.9 × 10^11^ (0.5 MGy), 2.8 × 10^11^ (1.0 MGy) and 1.8 × 10^12^ (5.0 MGy), at an energy of 12.3984 keV using a 10 × 15 µm beam. All datasets were processed with *XDS* (Kabsch, 2010*a* ▸,*b* ▸) and the resolution limit of each dataset was determined so that 〈*I*/σ(*I*)〉 in the highest shell was ∼2. Averaged resolution limits using nine crystals with error bars showing standard deviations are plotted as d*^2^ against the number of incident photons. Thaumatin was crystallized by the microseeding method based on the standard crystallization condition (Mueller-Dieckmann *et al.*, 2005 ▸).

The diffraction intensity of a crystal is proportional to the incident X-ray beam intensity and the number of unit cells irradiated by the incident X-ray beam. Fig. 2 ▸ shows the relationship between the number of incident photons and the obtained resolution limits of standard protein crystals (thaumatin crystals with sizes of ∼100 µm) measured on beamline BL32XU at SPring-8, Japan. The graph clearly demonstrates that the resolution improves as the crystal is exposed to more X-ray photons. Therefore, high-flux beamlines enable data collection at higher resolutions using a shorter exposure.

In the case of microcrystals, the smaller diffraction volume makes the diffraction intensities weaker than those of conventional larger crystals, and the noise introduced by background scattering becomes relatively large; therefore, maximization of the signal-to-noise ratio during data collection is quite important for successful structure determination (Holton & Frankel, 2010 ▸). Therefore, it is necessary to use a high-intensity microbeam with a size comparable to that of the target crystal (Nave, 1999 ▸).

### 2.2. Knowledge of the influence of radiation damage   

Although microbeams make it possible to record diffraction images from microcrystals, attention must be paid to radiation damage because the lifetime of a crystal is inversely proportional to the flux density. Radiation damage to protein crystals arises from two successive processes (O’Neill *et al.*, 2002 ▸). The primary process is induced by the initial absorption events of incident X-rays. The secondary process is caused by energetic electrons and radicals created *via* the primary process. The secondary process is the dominant cause of radiation damage in SR protein crystallography. As a result, two different kinds of radiation damage are observed in structural analyses: global and specific damage. Global damage is observed directly in diffraction images, while specific damage is a structural change that is observed as a change in electron density. Global damage includes intensity decay of diffraction spots, especially in higher resolution shells, and increases in crystal mosaic spread, unit-cell volume and Wilson *B* factors. Kmetko *et al.* (2006 ▸) reported that the relative *B* factor of a dataset depends linearly on the dose, with a gradient of 1 Å^2^ MGy^−1^. The acceptable dose regarding global damage at cryogenic temperatures appears to be essentially the same for every protein crystal. Owen *et al.* (2006 ▸) recommended a general maximum tolerable dose of 30 MGy. In contrast to global damage, specific radiation damage is sample-specific. Many types of specific damage have been reported, including the breakage of chemical bonds and the reduction of metalloproteins (Corbett *et al.*, 2007 ▸; Burmeister, 2000 ▸; Ravelli & McSweeney, 2000 ▸). These specific types of damage are well known to propagate faster than global damage (Holton & Frankel, 2010 ▸; Garman, 2010 ▸). The amount of radiation damage is proportional to both the flux density and the exposure time at the same photon energy. A higher flux density makes it more complicated to determine suitable exposure conditions in protein microcrystallography.

### 2.3. Sample-handling techniques   

In microcrystallography, crystals are sometimes too small to handle and mount individually. Additionally, multiple crystals are needed to complete a dataset as radiation damage prevents complete data collection using a single crystal. The solution to these problems is loading multiple crystals on a sample-mounting device.

A nylon or Kapton loop has been used as the conventional sample-mounting device. Although these conventional loops can scoop up a number of crystals at a time, recently more effective mounting devices composed of various materials with low background scattering have been developed; for example, thin films made of silicon (Zarrine-Afsar *et al.*, 2012 ▸; Mueller *et al.*, 2015 ▸; Roedig *et al.*, 2016 ▸), silicon nitride (Coquelle *et al.*, 2015 ▸), synthetic polymers (Huang *et al.*, 2015 ▸; Axford *et al.*, 2016 ▸; Baxter *et al.*, 2016 ▸; Schubert *et al.*, 2016 ▸) and graphene (Sui *et al.*, 2016 ▸). These devices have a large area, which facilitates the loading of a large number of crystals at the same time, and are designed to load crystals with a minimal amount of the surrounding mother liquor to reduce background scattering.

Some of these can be used for room-temperature data collection (Huang *et al.*, 2015 ▸; Roedig *et al.*, 2016 ▸; Axford *et al.*, 2016 ▸; Baxter *et al.*, 2016 ▸; Schubert *et al.*, 2016 ▸). One advantage of using these mounting devices is that they enable *in situ* data collection, which does not require the harvesting of crystals from the crystallization plate (Huang *et al.*, 2015 ▸; Schubert *et al.*, 2016 ▸). Another advantage is that some of the devices can trap microcrystals at prescribed positions, allowing serial crystallography with a high hit rate (Zarrine-Afsar *et al.*, 2012 ▸; Mueller *et al.*, 2015 ▸; Baxter *et al.*, 2016 ▸).

Another approach is the use of an injector, whereby a stream of microcrystals suspended in a suitable medium is flowed into the path of the X-ray beam. Botha *et al.* (2015 ▸) and Nogly *et al.* (2015 ▸) demonstrated the capability of sample injectors using lipidic cubic phase (LCP) as a matrix for microcrystal suspension. Meanwhile, Stellato *et al.* (2014 ▸) used a glass capillary through which a microcrystal suspension was continuously flowed.

Roessler *et al.* (2013 ▸) developed a sample-delivery method using a belt conveyer combined with an acoustic droplet ejection (ADE) system, in which small droplets containing microcrystals are translated to the X-ray beam position after being ejected onto the conveyer belt by the ADE. This method has recently been applied to time-resolved experiments using SFX (Fuller *et al.*, 2017 ▸).

## 3. Development of the microbeams required for microcrystallography   

As previously mentioned, a highly brilliant X-ray microbeam is optimal to measure diffraction data from microcrystals. A number of such high-brilliance beamlines dedicated to microcrystallography have been developed and small-size beams are now routinely available to crystallographers.

In 2005, the first 1 µm focused X-ray beam became available at the ID13 beamline at the European Synchrotron Radiation Facility (ESRF), France (Riekel *et al.*, 2005 ▸). This success accelerated the research and development of microfocusing techniques at SR facilities. Based on their experience, Flot *et al.* (2010 ▸) constructed the ID23-2 beamline at the ESRF and achieved a 7.5 µm focused beam with a photon flux of 4 × 10^11^ photons s^−1^. Meanwhile, at the 23-ID-B GM/CA-CAT beamline at the Advanced Photon Source (APS), USA (Yoder *et al.*, 2010 ▸), the minibeam concept was developed to realise a beam of 7.8 × 6.3 µm in size with a photon flux of 1 × 10^11^ photons s^−1^ (Xu *et al.*, 2011 ▸). In this design concept, a micrometre-sized beam is achieved by placing a micrometre-sized collimating pinhole in the path of a 100 µm beam. Changing the aperture size enables ready access to various beam sizes without the need to adjust other optical elements. The minibeam concept is more versatile to cover wide-ranging scientific targets, and there are now many beamlines using this design concept (Grochulski *et al.*, 2011 ▸; Hasegawa *et al.*, 2013 ▸).

**Figure 3:**
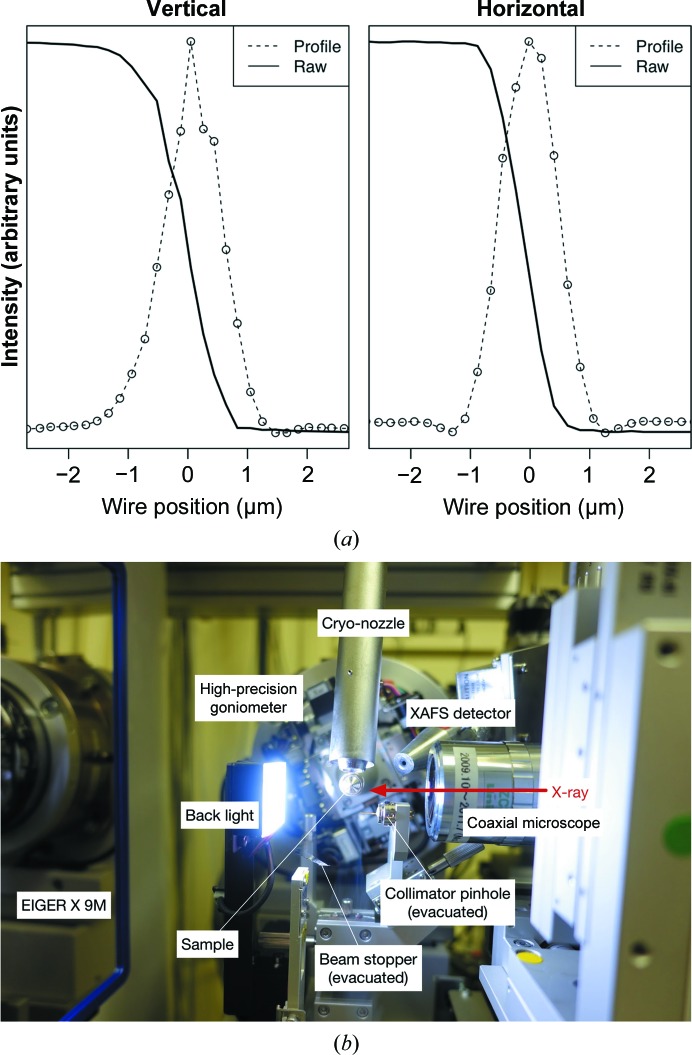
(*a*) Beam profiles of the microbeam BL32XU at SPring-8. A gold wire of 200 µm in diameter was used in the knife-edge scanning method (Mimura *et al.*, 2007 ▸). The wire was scanned with translation axes with 10 nm positioning accuracy and X-ray signals were detected with a PIN photodiode. (*b*) Photograph of the experimental station of BL32XU.

Beamline BL32XU (Fig. 3 ▸) was constructed at SPring-8 in 2009 (Hirata *et al.*, 2013 ▸). To achieve a focused microbeam, the surfaces of the focusing mirrors arranged in Kirkpatrick–Baez geometry were fabricated with atomic-scale accuracy by elastic emission machining (Yamauchi *et al.*, 2002 ▸). The beam size can be changed up to 10 µm (H) × 15 µm (V) and down to 0.9 µm (H) × 0.9 µm (V). The photon flux density, ∼10^10^ photons s^−1^ µm^−2^, is almost identical over the beam-size range. If a smaller beam with higher flux is required, 2 × 10^12^ photons s^−1^ with a 0.9 µm beam can be provided by changing the configuration of the optics (the profile is shown in Fig. 3 ▸).

At PETRA III (DESY, Hamburg, Germany), there are three microfocus beamlines, P11, P13 (Cianci *et al.*, 2017 ▸) and P14, and pioneering work on SSX was performed at P14 by Gati *et al.* (2014 ▸). Construction of beamlines dedicated to microcrystallography is currently in progress at many SR facilities, including new SR facilities such as NSLS-II, Brookhaven, USA and MAX IV, Lund, Sweden (Smith *et al.*, 2012 ▸; Fuchs *et al.*, 2014 ▸).

## 4. Effective data collection with a microfocused beam   

### 4.1. Multi-point data collection from a single crystal   

Because of their limited diffraction volume, the utilization of a brilliant microbeam is essential for data collection from microcrystals. Conversely, because the lifetime of each diffraction volume of a protein crystal is limited to a maximum dose of 20–30 MGy, excessive X-ray exposure causes serious radiation damage. To obtain a high-resolution complete dataset, it is important to choose an optimal data-collection method to collect diffraction signals from the limited diffraction volume under the dose limit.

**Figure 4:**
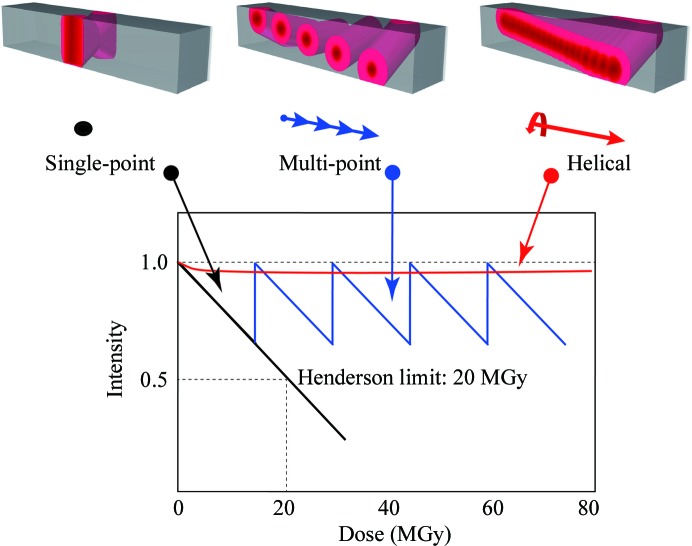
Schematic drawing of data-collection strategies and each change of diffraction intensity from a single crystal using a microbeam. The total image number in single-point data collection is limited by radiation damage. Multi-point data collection can increase the total image number by avoiding serious radiation damage by using a fresh crystal volume with translation of the irradiation point. Helical data collection enables an equal distribution of radiation damage over the entire crystal volume. Thus, the helical method gives completely smoothed scales and *B* factors for frames, which finally improves the data quality.

Pioneering structure analyses of GPCR crystals were achieved by combining *in meso* crystals with the minibeam of the 23-ID GM/CA-CAT beamlines at APS and the microfocus beam of the ID13 and ID23-2 beamlines at ESRF. The first structure analysis of a GPCR, excluding rhodopsin, was the human β_2_-adrenergic GPCR (Rasmussen *et al.*, 2007 ▸). Datasets were collected at 3.4 Å resolution using a single crystal with dimensions of 300 × 30 × 10 µm. It was stated that ‘microbeams were essential to obtain a favorable signal-to-noise ratio from the weakly diffracting thin crystals’ (Rasmussen *et al.*, 2007 ▸). The X-ray irradiation position was changed during data collection so as to avoid severe radiation damage. A small wedge of data at each position, typically 5–10°, was collected before marked radiation damage was observed (Rasmussen *et al.*, 2007 ▸). This multi-point data collection from a single crystal is powerful when the crystal size is larger than the beam size. In the multi-point method, however, the scale/*B* factors of the frames may show severe discontinuities when the irradiation point is changed depending on the accumulated damage (Fig. 4 ▸). The dose for each irradiation point should be estimated carefully to keep the radiation damage at an irradiation point under the dose limit, as in the single-point data-collection method. Flot *et al.* (2010 ▸) developed a helical data-collection strategy at ESRF beamline ID23. A full dataset was collected from a single crystal by continuously changing the irradiation point. For example, a 10 µm focused beam is translated along a crystal in 1 µm steps after collecting one diffraction image. The distance between irradiation points is normally set to be shorter than the beam size or the propagation length of radiation damage. The propagation length of radiation damage is known to be longer than the beam size because of photoelectron escape (Sanishvili *et al.*, 2011 ▸). This is different from the multi-point strategy, in which the distance between irradiation points is larger than the beam size or the propagation length of radiation damage. Flot and coworkers showed that this method enabled an equal distribution of radiation damage over the entire volume of the crystal. Thus, the helical method gives completely smoothened scales and *B* factors of frames, which finally improved the data quality. At BL32XU, the microfocus beamline at SPring-8, the helical method has been adopted as the standard method for data collection when the crystal size is larger than 10 µm.

Especially in helical data collection, estimating the correct absorbed dose is complicated because all of the experimental parameters, such as the beam size, X-ray energy, step length and desired amount of rotation range, need to be taken into account. *RADDOSE*-3*D* (Zeldin *et al.*, 2013 ▸) is a good program to estimate the absorbed dose and includes an option for helical data collection. At BL32XU at SPring-8, *KUMA* was developed to estimate the absorbed dose for helical data collection (Hirata *et al.*, 2016 ▸). *KUMA* estimates the absorbed dose from the beam size, the X-ray energy and the distance between irradiation points in helical data collection (Hirata *et al.*, 2016 ▸). It suggests exposure conditions with an absorbed dose of 10 MGy. A major difference between *KUMA* and *RADDOSE*-3*D* is that *KUMA* estimates the dose based on experimental data for the propagation length of radiation damage in frozen protein crystals. This program is also useful for multi-point data collection.

**Figure 5:**
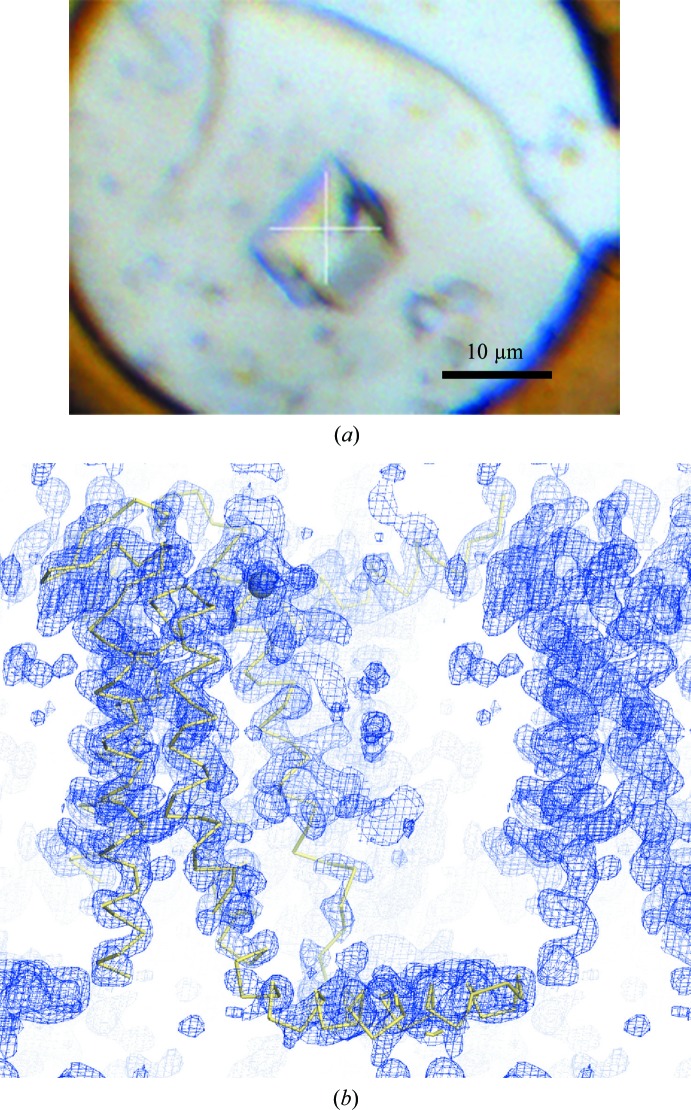
A single *in meso* microcrystal sufficient for structure solution by two-wavelength Hg-MAD at BL32XU. (*a*) A crystal of Hg-YidC. (*b*) The initial MAD-phased electron density at 1.0σ with a backbone of the final model of YidC. This was obtained by the helical data-collection method.

One of the successful experiments at BL32XU using *KUMA* was two-wavelength MAD phasing from a Hg-soaked *in meso* crystal of the membrane-protein insertase YidC (Kumazaki *et al.*, 2014 ▸; Fig. 5 ▸). Two datasets at 3 Å resolution covering a 360° oscillation range were collected from a single 13 µm cube-shaped crystal using the helical method at peak/edge wavelengths. A total of 12 irradiation points were used on the crystal at 1.0 µm intervals with a line-focused beam of 1.0 µm (H) × 15 µm (V). For each dataset, 12 frames with 2.5° oscillation (30° in total) were collected from each irradiation point. The step between irradiation points was shorter than the propagation length of radiation damage, 2.5 µm (FWHM), measured at 12.4 keV (Hirata *et al.*, 2016 ▸). Using these parameters, the maximum dose to the crystal was estimated using *KUMA* and finally set to 6–7 MGy for each data collection. The initial phase of YidC was determined from the single crystal (Fig. 5 ▸).

Equal distribution of precisely controlled radiation damage during helical data collection with a microfocused beam has shown good capabilities for samples that are difficult to phase (Nishizawa *et al.*, 2013 ▸; Tanaka *et al.*, 2013 ▸).

### 4.2. Multiple-crystal strategy   

One of the difficulties in microcrystallography is that the diffracting power of a single crystal may not be sufficient for complete and high-resolution data collection. High-resolution crystal structures of an engineered human β_2_-adrenergic receptor determined at APS (Cherezov *et al.*, 2007 ▸) and of cypovirus polyhedra at the Swiss Light Source (SLS; Coulibaly *et al.*, 2007 ▸) were pioneering works in improving the resolution using the multi-crystal strategy. In the β_2_-adrenergic receptor structure determination, using a 10 µm beam at beamline 23-ID-B GM/CA-CAT at APS, data collection was conducted using more than 40 cryocooled crystals with dimensions of around 30 × 15 × 5 µm. Datasets from a small wedge of 10–20° were collected from each crystal, and the best 27 datasets were merged into one complete dataset. The absorbed dose of each crystal would be 8–16 MGy after data collection when the beam size was 8 × 6 µm through a 10 µm aperture with 1 × 10^11^ photons s^−1^ flux, based on Sanishvili *et al.* (2011 ▸).

In this strategy, increasing the number of photons per oscillation angle can improve the resolution limit of each dataset at the expense of the completeness and redundancy of each dataset.

#### 4.2.1. Efficient finding of many microcrystals   

Mounted microcrystals are too small for their precise crystal positions in the loops to be recognized by an optical microscope. In particular, LCP crystals under cryogenic conditions are hidden in the LCP medium, which makes the recognition of crystals difficult (Cherezov *et al.*, 2009 ▸). Cherezov and coworkers reported a raster-scan method to find tiny crystals in the LCP medium utilizing a microbeam. To maximize the experimental efficiency, a two-step raster scan protocol was developed. In the first step, a large beam (∼100 × 40 µm) was used to roughly estimate the crystal position. A higher resolution raster scan with a smaller beam was then conducted in the area where diffraction spots were observed by the larger beam. After defining the crystal position with a precision of 5 µm, the procedure was repeated at a rotation angle of 90°. Hilgart *et al.* (2011 ▸) developed new software for efficient raster scanning of and data collection from microcrystals at APS. This program uses the automatic Bragg spot-finding program *DISTL* (Zhang *et al.*, 2006 ▸) to identify crystals from multiple raster-scanned images. Raster scanning demands the use of a microfocused beam to enhance the signal-to-noise ratio to find microcrystals. The most important specification for faster data collection is to use a fast-readout detector. The PILATUS/EIGER series of pixel-array detectors from DECTRIS (Brönnimann *et al.*, 2002 ▸; Casanas *et al.*, 2016 ▸) and the high-speed CCD detector MX-HS from Rayonix can be used for rapid raster scanning; for example, at a frame rate of 50 Hz at SPring-8 or 100 Hz at SLS (Wojdyla *et al.*, 2016 ▸). The program *Cheetah*, which was initially developed for hit finding in serial femtosecond crystallography (Barty *et al.*, 2014 ▸), can be applied to protein microcrystallography at SR facilities (see, for example, Nogly *et al.*, 2015 ▸). Parallel processing using multiple CPU cores has enabled on-the-fly analysis of raster-scan results.

#### 4.2.2. Automation of the multicrystal strategy   

**Figure 6:**
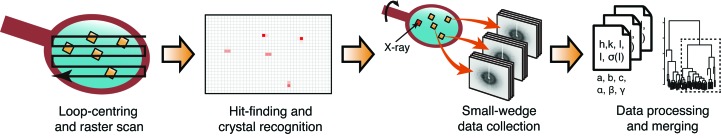
Automated microcrystal data-collection process. Firstly, the cryoloop is automatically centred and aligned under an optical microscope, followed by a low-dose X-ray raster scan in the defined area. The crystal positions are recognized by analyzing diffraction spots. A small-wedge diffraction dataset is collected from each crystal and processed to the merging of datasets to obtain complete and consistent data.

In the multicrystal strategy, it is important to collect datasets using as many crystals as possible. This procedure can therefore be highly demanding in terms of human input. As the procedure for the multicrystal strategy was established (Fig. 6 ▸), automation was desired to allow complete datasets to be obtained quickly and effectively. Recently, a fully automated data-collection system dedicated to the multicrystal strategy has been developed at the ESRF (Zander *et al.*, 2015 ▸). This procedure involves locating the positions of many cryocooled crystals mounted on a sample holder, predicting and ranking their relative diffraction strengths, and small-wedge data collection from each crystal. Hierarchical cluster analysis of intensities (Giordano *et al.*, 2012 ▸) was used to find the best combination of partial datasets to merge to give a complete dataset. At BL32XU, the automated data-collection system *ZOO* has recently been developed (Hirata *et al.*, in preparation). *ZOO* also allows fully automated data collection and data processing from multiple crystals. Automatic data-collection systems help to improve the resolution limit by increasing the number of photons per oscillation angle by using multiple crystals. Further development of automated systems, including the recognition of crystal size, to construct individual strategies will make multicrystal data collection more effective in the future.

### 4.3. The potential of serial synchrotron crystallography (SSX)   

**Figure 7:**
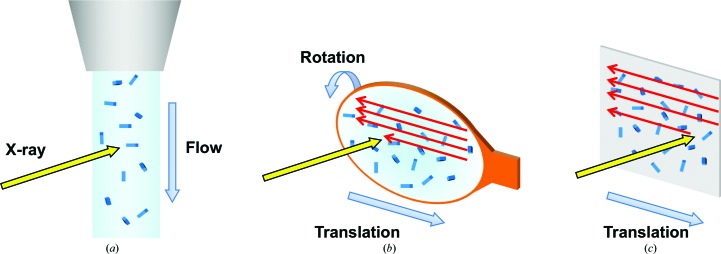
Various sample-delivery methods for serial synchrotron crystallography. (*a*) Liquid stream of a crystal suspension (Botha *et al.*, 2015 ▸; Nogly *et al.*, 2015 ▸), (*b*) loop-harvested cryocooled microcrystals (Gati *et al.*, 2014 ▸; Hasegawa *et al.*, 2017 ▸) and (*c*) thin-film substrate on which microcrystals are loaded (Coquelle *et al.*, 2015 ▸).

When a crystal is too small, small-wedge data collection may become difficult for two reasons. Firstly, the number of images that can be collected from one crystal is limited, and secondly, the two-dimensional raster scanning used to identify the crystal position can cause severe radiation damage before data collection. In such cases, serial crystallography is an alternative choice as an efficient data-collection method. In serial crystallography, a series of single-shot diffraction images are collected from one crystal or one crystal volume. The success of SFX using XFELs inspired researchers to establish SSX at synchrotron microfocus beamlines. To date, various sample-delivery methods for SSX have been examined (Fig. 7 ▸).

One advantage of SSX over SFX is that the typical exposure time of milliseconds to seconds allows the rotation of crystals during exposure, while only still snapshots are recorded in the SFX approach. Therefore, increases in the precision and efficiency of data collection can be expected. The first example of SSX was the work of Gati *et al.* (2014 ▸), who scooped up a number of microcrystals of TbCatB with a nylon loop and collected data at cryogenic temperature by using serial helical line scans to fully explore the region of interest, while shooting voxels of the sample independent of whether or not there was a crystal. Hasegawa *et al.* (2017 ▸) examined the advantage of single-shot rotation images and determined the optimum rotation step in SSX using mercury-derivatized microcrystals of luciferin-regenerating enzyme (LRE). Following the protocol of Gati *et al.* (2014 ▸), they collected seven datasets with different rotation steps ranging from 0 to 2.0° per frame. They processed the images individually and showed that the number of images required for successful Hg-SAD phasing was greatly decreased by rotation. The highest resolution was achieved at a rotation of 0.25° when datasets with the same number of images were compared. Although the optimum rotation step would depend on the sample, their results clearly demonstrated the advantage of rotation in SSX. Instead of rotation, the use of a nonmonochromatic pink beam has been proposed for the efficient coverage of reciprocal space and is planned at various beamlines (Stellato *et al.*, 2014 ▸).

Because crystals in SSX are influenced by radiation damage, the data-collection conditions need to be carefully controlled. Gati *et al.* (2014 ▸) compared the structure of TbCatB determined by SSX with that determined by SFX, which showed that the reduction of a disulfide bond was observed in the SSX structure, whereas it was not in the SFX structure. Hasegawa *et al.* (2017 ▸) also examined that influence of radiation damage in SSX using microcrystals of Hg-derivatized LRE. Their results demonstrated that an accumulated dose of above 1.1 MGy caused apparent specific damage at the Hg site, but improvements in resolution and anomalous signal were observed up to 3.4 MGy because of a higher signal-to-noise ratio.

One of the possibilities of SSX is room-temperature (RT) data collection from microcrystals. In the past two decades, most MX data collection has been performed at cryogenic temperature to mitigate radiation damage. However, the importance of RT data collection has recently come the fore because it may allow the observation of structural features which are normally hidden at cryogenic temperatures (Fraser *et al.*, 2011 ▸). Moreover, time-resolved (TR) experiments can be performed at RT. The issue with data collection at RT is the 70-fold more rapid progress of radiation damage compared with that at cryogenic temperature (Nave & Garman, 2005 ▸). Therefore, the fast sample exchange of SSX is advantageous for RT microcrystallography.

Stellato *et al.* (2014 ▸) performed RT SSX using a flow of suspended microcrystals through a capillary and determined the structure of lysozyme, whereas Botha *et al.* (2015 ▸) used a high-viscosity extrusive sample injector and demonstrated the feasibility of structure determination using SIRAS and/or MIRAS. Nogly *et al.* (2015 ▸) also used an LCP injector for the structure determination of bacteriorhodopsin (bR) at a resolution of 2.4 Å and revealed that bR adopted a slightly different structure to that obtained under cryogenic conditions.

Radiation damage in RT SSX was examined by Coquelle *et al.* (2015 ▸). They collected two datasets using a microbeam and a nanobeam with a dose per image of 3.2 and 29.1 MGy, respectively. They concluded that damage to the structure was limited and that the structural information was not compromised based on the observation that the disulfide bridges in the crystals were not broken in either case. Even though the flux density of the nanobeam was ten times that of the microbeam, the nanobeam did not cause more damage than the microbeam. They speculated that this was the result of a lag phase (Owen *et al.*, 2014 ▸) caused by the higher dose rate of the nanobeam experiment. Although further experiments are needed to confirm that this is the case, it is an intriguing result that indicates the feasibility of RT nanocrystallography.

The viability of TR SSX was demonstrated by Schubert *et al.* (2016 ▸). They collected 20 diffraction images covering 20° with exposure of 40 ms per frame using a newly developed *in situ* crystallization method. By merging datasets at an identical time interval from 46 crystals, they obtained 20 complete datasets at a time interval of 40 ms, which depicted the propagation of site-specific damage to disulfide bonds.

## 5. Data processing for multiple crystals   

### 5.1. Multicrystal data collection   

Data processing and merging multiple small-wedge datasets requires special care. Selecting frames without severe radiation damage, clustering of isomorphous data and the rejection of rogue data are important. Several protocols to merge multiple datasets from multiple crystals have been reported. Hierarchical cluster analysis (HCA) has been used to cluster isomorphous data. Giordano *et al.* (2012 ▸) used correlation coefficients (CCs) calculated from the common unique intensities of data-set pairs as similarities between datasets. However, CCs may not be available in the case of smaller wedges and lower crystal symmetry. The *BLEND* software (Foadi *et al.*, 2013 ▸) performs HCA based on the similarity of unit-cell parameters, which is a necessary condition for isomorphism. *BLEND* not only performs HCA, but also performs scaling and merging using *AIMLESS* after the rejection of frames suffering from intensity decay caused by radiation damage (Axford *et al.*, 2015 ▸). Assmann *et al.* (2016 ▸), rather than classifying datasets prior to merging, identified non-isomorphous datasets in the merging step, which deteriorate the average value of CC_1/2_ in resolution shells. Another approach that directly optimizes data-quality indicators of merged intensities such as *R* factors and CC_1/2_ was employed by Zander *et al.* (2016 ▸). They used a genetic algorithm to find the optimal combinations of datasets.

### 5.2. Serial crystallography   

The data-processing protocol in SSX depends on whether or not the crystals are rotated. When multiple still snapshots are collected, data-processing software developed for SFX, such as *CrystFEL* (White *et al.*, 2012 ▸, 2016 ▸) and *cctbx.xfel* (Sauter *et al.*, 2013 ▸), can be used (Botha *et al.*, 2015 ▸; Nogly *et al.*, 2015 ▸; Coquelle *et al.*, 2015 ▸). These program suites have functionalities including spot finding, indexing, integration and merging. Currently, Monte Carlo integration is the most routinely used method to merge observations from several crystals. Conversely, when the sample is rotated during exposure, well established data-processing software for conventional rotation data such as *XDS* (Kabsch, 2010*a* ▸,*b* ▸) can be used. For example, Gati *et al.* (2014 ▸) grouped consecutive images recorded from the same crystal using *CrystFEL*. Each group was indexed and integrated by *XDS* using three-dimensional profile fitting, and the integrated intensities were then scaled and merged using *XSCALE* (Kabsch, 2010*a* ▸,*b* ▸). Hasegawa *et al.* (2017 ▸) also used *XDS* for data processing, but they treated individual images as random snapshots. In this process, the full reflection intensities were estimated using single frames. The integrated intensities were then averaged after applying a linear scale factor to each image. They demonstrated that discarding reflections with lower partialities played an important role in obtaining more accurate data. One important development in SSX data collection and data processing would be on-the-fly data processing to allow real-time monitoring of completeness, 〈*I*/σ(*I*)〉 and CC_1/2_. These parameters could be used to provide feedback on the experimental conditions and/or when to make the decision to finish data collection.

## 6. Summary and future prospects   

The *in meso* method for crystallization of membrane proteins was developed in the late 1990s (Caffrey, 2003 ▸). At the same time, microfocus beamlines were introduced for MX. In addition to these two innovations, various advances in data-collection strategies in microcrystallography have enabled structure determination from a single microcrystal with a size of around 10 µm. Moreover, technological developments such as SFX and SSX for data collection from micrometre-sized to submicrometre-sized crystals were stimulated. The optimal data-collection strategy depends on the size of a crystal and its sensitivity to radiation damage.

Single-crystal data collection with the helical method is suitable for crystals with edge sizes of a few tens of micrometres and is effective for initial phase determination without the problem of non-isomorphism between crystals and severe radiation damage. Helical data collection is a powerful method for needle-like crystals.

When the size of the crystal is less than 10 µm or its diffracting power is too weak, data collection using multiple crystals should be considered to accumulate diffraction signals. Automated data-collection pipelines for multiple crystals have been developed to reduce human input, such as crystal centring and considerations of the data-collection strategy for each crystal. Multicrystal data collection is now feasible for a few tens to several thousands of microcrystals. The accumulation of a large quantity of diffraction data by multicrystal data collection provides the possibility of improving the resolution limit. However, care needs to be taken for crystals that are sensitive to radiation damage because the raster scanning employed for crystal centring uses X-rays as a probe. In such cases, serial crystallography (SSX or SFX using SR or an XFEL, respectively) is another option. Moreover, serial crystallography is suitable for studies of structural dynamics. SFX has been extended to time-resolved experiments using the pump-and-probe technique. Fruitful results have already been obtained such as time-resolved studies of photosystem II (Young *et al.*, 2016 ▸; Suga *et al.*, 2017 ▸), photoactive yellow protein (Pande *et al.*, 2016 ▸) and bacterio­rhodopsin (Nango *et al.*, 2016 ▸), and in addition a time-resolved study using mix-and-inject SFX (Stagno *et al.*, 2016 ▸). Meanwhile, SSX can be used to collect diffraction data at room temperature. In SSX, the use of a pink beam is being studied and it is possible to irradiate a sample with a flux that is several tens of times more powerful by making a pink microbeam (Stellato *et al.*, 2014 ▸).

In addition to the construction of new SR facilities such as NSLS-II and MAX IV, the upgrading of existing SR facilities, for example ESRF-II, APS-U, SPring-8 II and PETRA IV, to low-emittance rings aiming at higher brilliance is planned. The development of microbeams at SR beamlines will continue to lead to smaller beam sizes with higher flux. Along with development of the light sources, advances in measurement technology at MX beamlines will enable a further reduction of measurable crystal size from several micrometres to sub­micrometre. The time resolution in time-resolved experiments will be improved from millisecond to submillisecond. Meanwhile, the reduction of the measurable crystal volume means a decrease in the copy number of unit cells, and the use of scattering signals recorded between diffraction spots should come into view for submicrometre crystals, which can be regarded as a cluster of proteins of about 10^6^ unit cells (Spence *et al.*, 2011 ▸). If it becomes possible to measure the diffraction image from such submicrometre crystals using future ultrahigh-brilliance microbeams, then the phase problem will be solvable by applying the analytical method of X-ray coherent diffraction imaging. This will advance protein crystallography to the next stage.

The high brilliance of SR beamlines and advances in measurement technology will lead to further progress in protein microcrystallography.
